# *Cladosporium cladosporioides* Fungemia in a Patient with Non-Hodgkin Lymphoma: An Extremely Rare Case and Review of the Literature

**DOI:** 10.3390/reports9010060

**Published:** 2026-02-13

**Authors:** Denis Niyazi, Nikol Daskalova, Ilina Micheva, Temenuga Stoeva

**Affiliations:** 1 Clinical Microbiology Laboratory, University Hospital “St. Marina”—Varna, 9010 Varna, Bulgaria; temenuga.zhekova@mu-varna.bg; 2 Department of Microbiology and Virology, Medical University of Varna, 9002 Varna, Bulgaria; 3 Clinical Hematology Clinic, University Hospital “St. Marina”—Varna, 9010 Varna, Bulgaria; nikol.daskalova@mu-varna.bg (N.D.); ilina.micheva@mu-varna.bg (I.M.); 4 Hematology Section, Second Department of Internal Diseases, Medical University of Varna, 9002 Varna, Bulgaria

**Keywords:** *Cladosporium cladosporioides*, fungemia, immune suppression, non-Hodgkin lymphoma

## Abstract

**Background and Clinical Significance**: *Cladosporium cladosporioides* is a ubiquitous dematiaceous mold that is rarely implicated in invasive human diseases and often considered a saprophyte and plant pathogen. **Case Presentation**: We report an extremely rare case of *C. cladosporioides* fungemia in a 61-year-old man with mantle cell lymphoma undergoing chemotherapy. The patient developed fever, dry cough and cavitary pulmonary lesions in the setting of profound immunosuppression. Blood cultures yielded slow-growing dark pigmented mold after prolonged incubation and species identification was achieved using MALDI-TOF mass spectrometry with an alternative fungal database, supported by microscopic morphology. Serum 1,3-β-D-glucan levels were markedly elevated, while galactomannan antigen testing was negative. Antifungal susceptibility testing demonstrated activity of triazoles and targeted therapy with voriconazole led to clinical improvement. A review of the literature identified only five previously reported cases of invasive *C. cladosporioides* infections worldwide, involving primarily pulmonary and central nervous system disease. **Conclusions**: To the best of our knowledge, this is the first documented case of fungemia caused by this species. The current report highlights the pathogenic potential of *C. cladosporioides* in immunocompromised hosts, the diagnostic challenges posed by slow-growing dematiaceous fungi, the importance of prolonged culture incubation, fungal biomarkers and advanced identification techniques for timely diagnosis and management.

## 1. Introduction and Clinical Significance

*Cladosporium cladosporioides* is a filamentous fungus belonging to genus *Cladosporium*, which comprises more than 700 species [1]. It is a dematiaceous mold (produces dark pigment) with a ubiquitous distribution and is commonly found both indoors and outdoors. The spores of *C. cladosporioides* are present in soil, decaying organic matter, household dust and even food products [2]. Formerly, it was considered a saprophyte and primarily a plant pathogen associated with leaf blight [3], but there are increasing reports of its growing medical importance, mainly as an airborne allergen and sometimes as an opportunistic pathogen [4].

Inhalation and retention of *C. cladosporioides* spores in the respiratory tract are associated with a hypersensitivity reaction (primarily type I), and have been linked to allergic conditions such as rhinitis, asthma exacerbation and hypersensitivity pneumonitis [5]. These conditions are often seasonal, occurring predominantly during warm and humid months when spore abundance is highest. Studies involving mouse models that examine the role of *C. cladosporioides* in allergic pathology demonstrate that the fungus induces upregulation of inflammatory cytokines (IL-4, IL-5, IL-13), thereby mimicking asthma [6]. Occasionally, *C. cladosporioides* can cause localized or invasive infections, with immunosuppression being the main risk factor. In immunocompromised hosts, the mold infection may manifest as phaeohyphomycosis of the skin and underlying tissue, sinusitis, keratitis and pulmonary infection [7]. In patients with hematological malignancies (mainly lymphoma and leukemia), a unique combination of both disease-related immune defects such as neutropenia, impaired immune cell function, and treatment-related factors including immune cell depletion, bone marrow destruction and mucosal barrier injury predispose to invasive fungal infections (IFIs) [8].

Diagnosis and treatment of *C. cladosporioides* infection are difficult due to its innate resistance to certain antifungal agents, as well as due to the complex diagnostic approach, including various methods and techniques [9].

We present an extremely rare case of fungemia caused by *C. cladosporioides* in a patient with non-Hodgkin lymphoma and aim to provide a literature review of invasive diseases associated with *C. cladosporioides*.

## 2. Case Presentation

A 61-year-old man was admitted to the Hematology clinic of the University Hospital “St. Marina”, Varna, Bulgaria with complaints of fatigue. Two months prior to the current hospitalization, he had been urgently admitted to the Neurology clinic of the same hospital because of severe vertigo, gait instability, staggering and falls. Brain computed tomography showed no abnormalities, while laboratory blood tests revealed marked leukocytosis (640.7 × 10^9^/L) and anemia (hemoglobin 52 g/L; red blood cells 1.99 × 10^12^/L; hematocrit 0.21 L/L). After consultation with a hematologist, the patient was transferred to the Hematology ward for further diagnostic evaluation. A flow cytometry test demonstrated a pathological B-lymphocytic population and treatment with first-line bendamustine-based chemotherapy (70 mg/m^2^ on days 1 and 2) was initiated. Serological tests also confirmed chronic hepatitis B infection; thus rituximab was not prescribed. No comorbidities were disclosed by the patient. Despite frontline therapy, the disease demonstrated an inadequate response with no hematological response observed—persistent leukocytosis of 300–440 × 10^9^/L, consistent with refractory disease. Salvage chemotherapy with the BAC500 regimen (bendamustine 70 mg/m^2^ on days 1 and 2 and low-dose cytarabine 500 mg/m^2^ on days 2–4) was subsequently initiated.

Upon the current admission, the patient was in poor general condition, alert and afebrile, corresponding to an Eastern Cooperative Oncology Group (ECOG) performance status of 3, reflecting significant functional limitation attributable namely to the disease burden. The skin and mucous membranes were pale. No lymphadenopathy was detected. Lung auscultation was clear. Cardiac rhythm was regular, with blood pressure of 125/65 mmHg. The abdomen was soft and non-tender. Hepatosplenomegaly was present. No peripheral edema was observed.

Admission blood tests demonstrated leukocytosis (495.7 × 10^9^/L) with lymphocytosis (187.7 × 10^9^/L) and anemia (red blood cells 2.06 × 10^12^/L; hemoglobin 77 g/L). Several days after admission, the patient developed dry cough and fever. Imaging studies revealed cavitary lesions and mediastinal lymphadenopathy. Inflammatory markers were elevated—C-reactive protein (20.4 mg/L) and procalcitonin (0.083 ng/mL). Sputum could not be obtained for microbiological examination. Bronchoscopy was not performed due to the risk of pneumothorax and bleeding. Multiple sets of blood cultures were collected according to established protocol. Empirical antimicrobial therapy with piperacillin/tazobactam (3 × 4.5 g/d) was initiated. Bone marrow biopsy confirmed mantle cell lymphoma. The patient was stratified as high-risk according to the Mantle Cell Lymphoma International Prognostic Index (MIPI). A second course of bendamustine and cytarabine was administered; however, due to refractory disease, a decision was made to switch to ibrutinib (140 mg once daily). Due to a lack of clinical response to antimicrobial therapy, piperacillin/tazobactam was switched to meropenem (3 × 1 g/d).

**Figure 1:**
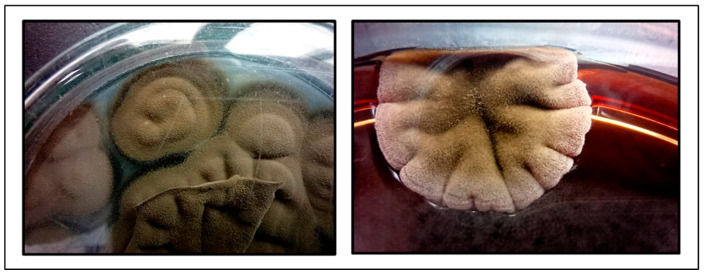
Fungal growth on Sabouraud dextrose agar and blood agar after 72 h cultivation at room temperature.

One aerobic blood culture flagged as positive but showed no growth after 24 h of incubation at 37 °C under aerobic conditions. Gram staining revealed no microbial cells. After prolonged incubation (72 h) at room temperature on Sabouraud dextrose agar (SDA) and blood agar, small, dark greenish, velvety colonies of mycelia appeared (Figure 1).

To exclude contamination, the blood sample was recultured and identical mycelial growth after 72 h was documented. Serum samples were obtained for *Aspergillus* galactomannan antigen (Platelia *Aspergillus* Ag, Bio-Rad Laboratories, Hercules, CA, USA) and 1,3-beta-D-glucan (FUJIFILM Wako, Neuss, Germany). Galactomannan antigen was negative (optical density index < 0.5), whereas 1,3-beta-D-glucan was significantly elevated at 116.8 pg/mL (normal range < 7 pg/mL). Based on these results, treatment with voriconazole (12 mg/kg on day 1 and 8 mg/kg from day 2) was initiated. 1,3-beta-D-glucan retesting after several days demonstrated still elevated levels (138.1 pg/mL).

**Figure 2:**
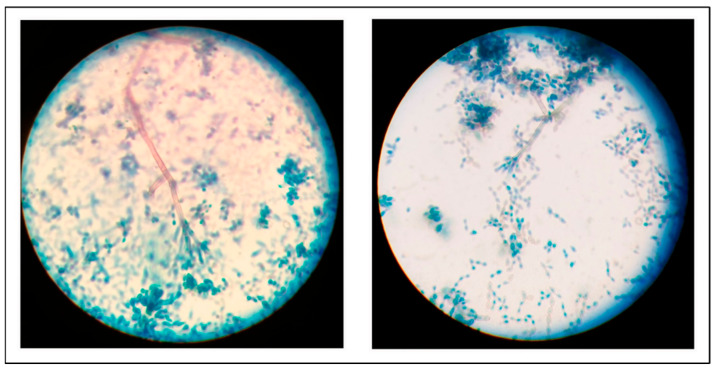
Dark pigmented conidiophores and stained conidia with lactophenol cotton blue (×1000).

A slide prepared from the mycelium using lactophenol cotton blue staining showed dark segmented conidiophores and stained conidia (Figure 2) suspicious for dematiaceous mold.

**Figure 3:**
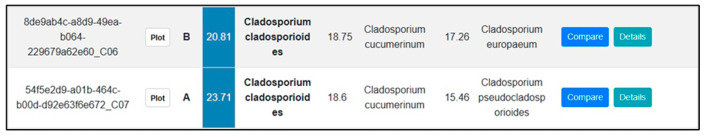
Identification by MALDI Biotyper and alternative database.

MALDI Biotyper (Bruker, Bremen, Germany) was used for further fungal identification. The fungal colonies underwent protein extraction according to Normand’s protocol [10] and while the general MALDI database could not identify the mold, the generated protein spectra were subjected to alternative database (https://msi.happy-dev.fr/) (accessed on 18 August 2025) testing which confirmed the mold as *Cladosporium cladosporioides* (Figure 3). Antifungal susceptibility testing by an E-test demonstrated the following results: itraconazole (0.75 µg/mL), voriconazole (1 µg/mL), micafungin (4 µg/mL) and anidulafungin (3 µg/mL). In the absence of official CLSI/EUCAST breakpoints for *Cladosporium* spp., the minimum inhibitory concentration upper end ECOFF values for *Aspergillus* spp. were used (EUCAST, 2025). Based on these findings and according to EORTC/MSGERC criteria, infection with *C. cladosporioides* was confirmed [11].

Under treatment with ibrutinib and voriconazole, normalization of peripheral blood counts was achieved. At the six-month reassessment, a partial response was documented, with significant reduction in splenomegaly and hepatomegaly. Additionally, the previously described cavitary pulmonary lesion demonstrated fibrotic transformation without signs of active infection.

### Literature Review

Using keywords (“*Cladosporium cladosporioides*” and “infection”) in the scientific databases of Scopus, PubMed and Clarivate, a total of 125 literature sources were found. Our inclusion criterion was *Cladosporium cladosporoioides* human invasive infection. Most of these reports were related to experimental models (non-real infections), plant-based (non-human) or non-invasive infections (allergic or superficial complications) and were excluded. Only five published cases of invasive *Cladosporium cladosporioides* infection were identified across different countries between 1975 and 2024 (Table 1). Patients ranged from 15 to 84 years old, with both sexes represented. Most patients had significant comorbidities or immunocompromising conditions. Infections affected either the brain (2 cases: India, The Netherlands) or the lungs (3 cases: Japan, Brazil, USA). Diagnosis commonly relied on culture and microscopy, with several cases also using DNA sequencing to confirm species identification.

Voriconazole and itraconazole were used in two (India, Japan) and one cases (Brazil) respectively, all with favorable outcome. Older reports (The Netherlands, USA) did not specify treatment [7,15]. The USA case resulted in a lethal outcome and the case from The Netherlands had no outcome reported. Generally, most recent cases responded well to modern antifungal therapy (especially voriconazole), whereas older cases had poorer documentation and outcomes [12,13].

To the best of our knowledge, the hereby presented case is the first documented fungemia caused by *Cladosporium cladosporioides*. Based on the imaging results, we can speculate that fungemia most probably originates from the respiratory tract complicated by angioinvasion.

## 3. Discussion

The reported case of *C. cladosporioides* fungemia highlights the diagnostic and therapeutic complexity of the invasive fungal infections (IFI) in patients with hematologic malignancies. The patient, a 61-year-old man with newly diagnosed mantle cell lymphoma, developed respiratory symptoms and persistent inflammatory response against a background of progressive immunosuppression including severe lymphoproliferative disease, repeated chemotherapy with a bendamustine and cytarabine regimen, and a chronic hepatitis B infection. These conditions constitute major predisposing factors for opportunistic fungal infections, particularly molds [16]. Bendamustine is unique among the chemotherapeutics because it combines both alkylating agent and purine-analog-like properties. This drug leads to profound and prolonged immunosuppressive effects, particularly on T-cell–mediated immunity, associated with neutropenia (when used in combination) and impaired antigen presentation, reduced cytokine signaling, and hypogammaglobulinemia when combined with anti-CD20 therapy—effects explaining the bendamustine strong linkage to opportunistic infections, including those caused by molds [17].

The radiologic findings of cavitary lung lesions and mediastinal lymphadenopathy raised the suspicion for IFI early in the course of the disease. Cavitary pulmonary lesions in patients with hematologic malignancies are associated with a wide differential diagnosis, encompassing infectious causes (bacterial and fungal) and noninfectious etiologies related to the underlying disease [18]. However, the inability to obtain sputum, combined with the high procedural risk of bronchoscopy, significantly limited the direct microbiological evaluation. This diagnostic challenge is frequently encountered in hematology patients where the patient’s general condition restricts access to optimal diagnostic procedures. As a result, the diagnostic approach is based on the combination of blood cultures, non-cultural methods, and indirect biomarkers [19].

In this case, repeated blood cultures eventually yielded growth of slow-growing, dark-green velvety mold after prolonged incubation at room temperature—features compatible with dematiaceous fungi. The reproducibility of growth in multiple growth media minimized the likelihood of contamination. It is known that isolation of molds from blood cultures, with few exceptions such as *Fusarium* spp., is often related to pseudofungemia (fungal isolation from one or more blood cultures in a patient with no clinical, laboratory and radiologic evidence of IFI, a finding attributable to a contaminated sample) [20,21]. To aid in distinguishing true fungemia from pseudofungemia, Duthie and Denning proposed diagnostic criteria incorporating the patient’s clinical history as well as relevant clinical and microbiological features. Although these criteria were originally developed for *Aspergillus* spp., they may be broadly applied to other molds, including *Cladosporium cladosporioides* [22]. Furthermore, the lack of microbial cells (spores or hyphae) in the sample when complex staining methods (such as Gram’s) are used can lead to false negative results due to fungal element distraction while heat-fixing the slide [23].

Although the galactomannan antigen test was negative, the significantly elevated 1,3-beta-D-glucan supported the presence of an invasive fungal infection, consistent with published data indicating that galactomannan has limited sensitivity outside *Aspergillus* infections, whereas 1,3-beta-D-glucan is broadly positive in infectious complications caused by dematiaceous molds, including *Cladosporium* spp. [24].

Species identification by MALDI Biotyper, supported by microscopic investigation, confirmed *Cladosporium cladosporioides*, a rare but increasingly recognized cause of invasive phaeohyphomycosis. Although typically environmental and of low pathogenicity, *Cladosporium* may cause severe and disseminated disease in immunocompromised hosts. Reported infections include pneumonia, skin and soft-tissue disease and central nervous system involvement [12,24,25]. The patient’s presentation with cavitary pulmonary lesions and fungemia aligns with these described patterns of severe IFI [26].

The elevated and repeatedly positive 1,3-beta-D-glucan values further strengthened the diagnosis. According to EORTC/MSGERC criteria [11], the combination of host factors (hematologic malignancy, chemotherapy), clinical criteria (cavitary pulmonary lesions), and mycological evidence (positive blood cultures for mold, positive 1,3-beta-D-glucan) is sufficient to define proven invasive mold infection which led to prompt initiation of mold-active antifungal therapy.

Voriconazole was selected as first-line treatment, which is consistent with existing literature showing variable but generally favorable susceptibility of *Cladosporium* spp. to triazoles [27]. The performance of E-test antifungal susceptibility further supported this choice. Given the organism’s intrinsic resistance to some agents and the limited clinical data available, individualized susceptibility testing is considered crucial.

Our case highlights several important aspects of managing IFIs in hematology patients: non-classic pathogens such as *Cladosporium* spp. should be considered when standard diagnostics are negative but clinical suspicion remains high; 1,3-beta-D-glucan is a valuable early biomarker in non-*Aspergillus* mold infections; extended incubation and culture at appropriate temperatures may be required to detect slow-growing dematiaceous fungi; early antifungal therapy, even empirically, is essential to improve outcomes, given the high mortality associated with IFI in immunocompromised hosts.

The presented overview of the *C. cladosporioides* invasive infections supports the potential of this widespread dematiaceous mold to cause severe complications in the human host, as well as to lead to fatal outcomes if not recognized and adequately treated.

## 4. Conclusions

In conclusion, we report a rare case of *Cladosporium cladosporioides* fungemia and suspected invasive pulmonary infection in a patient with mantle cell lymphoma. This report emphasizes the need for a high index of suspicion, careful interpretation of fungal biomarkers, and persistence in microbiologic testing to achieve a definitive diagnosis.

## Figures and Tables

**Table 1:** Literature review results on *Cladosporium cladosporioides* invasive infections.

Country	Year	Age	Sex	Underlying Disease	Site of Infection	Methods	Treatment	Outcome	Ref.
India	2024	15	M	Severe dengue and intracranial bleeding	Brain (abscess)	Culture, microscopy and Sanger sequencing of 18S rRNA	Voriconazole	Favorable	[12]
Japan	2023	84	F	Febrile neutropenia, endometrial cancer	Lungs (abscess)	Culture, microscopy and DNA sequencing	Voriconazole	Favorable	[13]
Brazil	2016	59	M	Not reported	Lungs (hemorrhagic pneumonia)	Culture and microscopy	Itraconazole	Favorable	[14]
The Netherlands	2002	30	M	Not reported	Brain	Culture, microscopy, DNA sequencing	Not reported	Not reported	[7]
USA	1975	58	F	Diabetes, hypertension, cerebrovascular accident	Lungs	Culture and microscopy	Not reported	Lethal	[15]

M—male; F—female.

## Data Availability

The original data presented in this study are available on reasonable request from the corresponding author. The data are not publicly available due to privacy concerns.

## Abbreviations

The following abbreviations are used in this manuscript:

MALDI	Matrix-Assisted Laser Desorption/Ionization
EORTC/MSGERC	European Organization for Research and Treatment of Cancer and the Mycoses Study Group Education and Research Consortium
IFI	Invasive Fungal Infection
